# Posterior reversible encephalopathy syndrome after postpartum hemorrhage and uterine artery embolization: A case report

**DOI:** 10.1097/MD.0000000000008973

**Published:** 2017-12-08

**Authors:** Fangfang Shi, Liwei Shen, Yonghui Shi, Lei Shi, Xiaoli Yang, Zhi Jin, Wenpeng Liu, Danhong Wu

**Affiliations:** Department of Neurology, Shanghai Fifth People's Hospital Affiliated to Fudan University, Shanghai, China.

**Keywords:** contrast-induced encephalopathy, posterior reversible encephalopathy syndrome, postpartum hemorrhage, uterine artery embolization

## Abstract

**Rationale:**

Posterior reversible encephalopathy syndrome (PRES) is characterized by clinical and radiological features, including headache, disturbed consciousness, seizures, and cortical blindness associated with findings indicating posterior leukoencephalopathy on imaging studies. Ours is the first case of PRES developing after postpartum hemorrhage and uterine artery embolization.

**Patient concerns:**

An 18-year-old patient had postpartum hemorrhage after a normal delivery. She required uterine artery embolization to stop the bleeding; however, she developed PRES 2 hours after the surgery.

**Diagnoses:**

Brain computed tomography suggested subarachnoid hemorrhage or cerebral venous sinus thrombosis. However, findings on magnetic resonance imaging were highly indicative of PRES.

**Interventions:**

The patient received diazepam and midazolam to prevent seizures.

**Outcomes:**

Seizures were controlled on the first day. The patient's visual acuity returned to normal on the fourth day of admission. Thirteen days after admission, her neurological signs and symptoms were completely managed.

**Lessons:**

PRES may be related to postpartum hemorrhage, blood pressure fluctuation, inflammation, and contrast agents. Collectively, they cause a breakage in the blood–brain barrier and endothelial cell damage, eventually leading to PRES. We also found PRES had many features similar with contrast-induced encephalopathy.

## 1 Introduction

Posterior reversible encephalopathy syndrome (PRES) is a disease characterized by clinical and radiological features, including headache, altered mental functioning, seizures, and cortical blindness associated with findings indicating posterior leukoencephalopathy on imaging studies.^[1–2]^ It was first described by Hinchey^[1]^ in 1996 and was originally called posterior reversible leukoencephalopathy syndrome. The name of the disease remains controversial, with many people believing the name is inaccurate. However, for nearly 20 years, it has been known as PRES.^[2]^

PRES always presents in patients with preeclampsia/eclampsia, hypertensive blood pressure, immunosuppressive therapy after transplantation, antitumor therapies, renal disease, sickle anemia, spinal cord injury, and even snake bites.^[3–13]^

A recent case showed that postpartum hemorrhage, without high blood pressure, can lead to PRES.^[14]^ Herein, we present a case of PRES that developed 2 hours after uterine artery embolization for preventing massive uterine bleeding. Here, we aimed to determine to the causative factors for PRES. Is PRES related to iodine contrast agent or did the patient experience contrast-induced encephalopathy (CIE)?

### 1.1 Patient information

Approval for the study by the local institutional review board was waived because it was a case report. An 18-year-old pregnant woman at 40 weeks gestation presented to our hospital at 8:00 on January 12, 2017 with vaginal discharge and abdominal pain, which started an hour previously.

### 1.2 Clinical findings

At the time of admission, her cervix was dilated 2 cm. Soon after, her uterus contracted once for 25 seconds every 4 to 5 minutes. The contraction was weak, and the fetal heart rate was good. At that time, her blood pressure was 135/85 mm Hg. The placenta from the cervix mouth was over 70 mm, and the fetal umbilical cord coiled around the neck.

Fetal weight was reassessed and was found to be approximately 4 kg, which was higher than that initially thought; thus, she was recommended for caesarean section. However, the patient and her family refused the recommendation and chose vaginal delivery. Approximately 6 hours later (14:11, January 12), the mother gave birth to a live female baby weighing 4.9 kg. The mother appeared stable postpartum. Her face was pink, and she did not complain of any discomfort. Her blood pressure was 135/90 mm Hg at that time.

**Figure 1:**
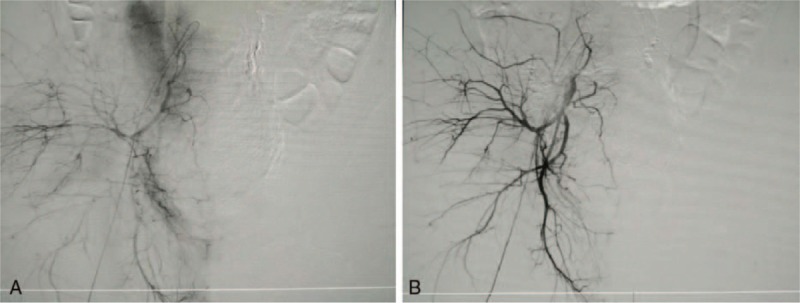
(A) Patient was bleeding from the uterine artery. (B) The bleeding was stopped.

However, after the delivery, she hemorrhaged and lost approximately 1500 mL of blood. Her doctors immediately injected 20 U oxytocin and placed an intrauterine balloon catheter to stop the uterine bleeding. Then, 5 units of red blood cell suspension, 400 mL of blood plasma, and 5 units of cryoprotein were administered to stop the bleeding. After, the patient vomited, and her bleeding continued (15:10, January 12). Her blood pressure was 145/92 mm Hg. Doctors previously informed interventional physicians of her state; however, they were occupied with another surgery. Finally, 2 hours after her labor (16:20, January 12), she was wheeled to the interventional room to undergo uterine artery angiography and embolization surgery (Fig. 1). Surgery (17:25, January 12) successfully halted the bleeding, and the patient did not report any postoperative discomfort.

One hour after the operation (18:30, January 12), the patient suddenly developed a twitch in her left periocular muscles. Her blood pressure was 160/100 mm Hg. Her doctor prescribed oxygen therapy and 10 mg nifedipine. By 18:34, the patient developed a twitch in both her left and right periocular muscles, following which she began to spasm and became unconscious. Her doctor immediately prescribed 2 doses of 10 mg venous diazepam, 125 mL mannitol, and 4 g magnesium sulfate. Her spasms slowly ceased, and she regained consciousness. However, the patient soon complained of binocular blindness and nausea (18:40, January 12). Her blood pressure was 162/104 mm Hg. Twenty-five minutes after the blindness developed (19:05, January 12), the patient gradually lapsed into unconsciousness again.

**Figure 2:**
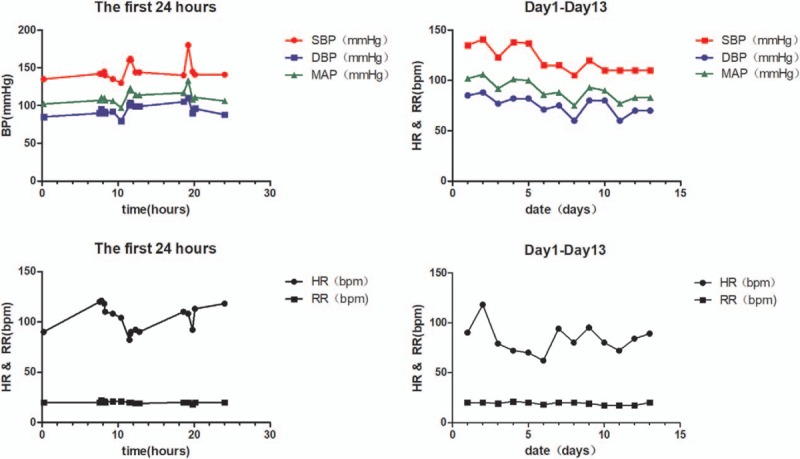
The fluctuation of vital signs.

We recorded her vital signs including blood pressure (BP), heart rate (HR), and respiratory rate (RR). The changes were shown by line graphs (Fig. 2).

### 1.3 Diagnostic assessment

**Figure 3:**
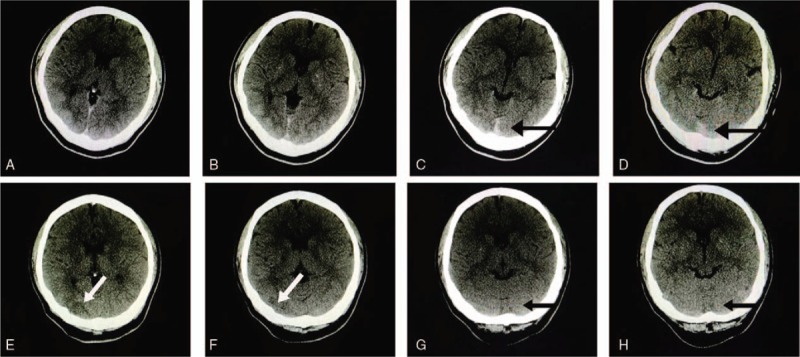
(A) Higher-density signal than (E) on the right occipital lobe. (B) Higher-density signal than (F) on the right occipital lobe. (C, D) High-density signals in the posterior fossa on the evening of January 12, 2017. (G, H) The high-density signals in the posterior fossa disappeared on the morning of January 13, 2017.

**Figure 4:**
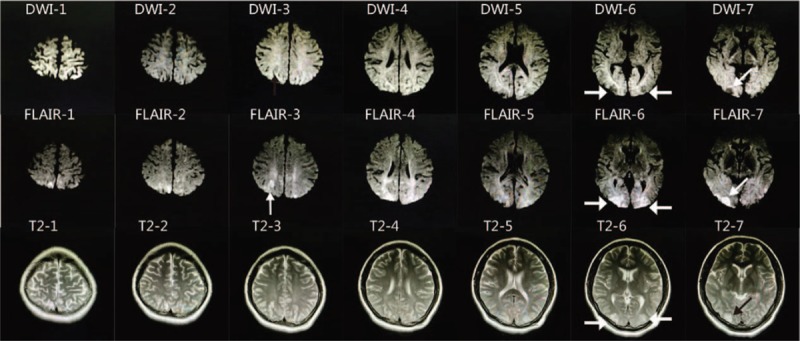
(DWI-6/7) Low-sensitivity signals on the bilateral occipitoparietal cortex; (flair-6/7) high-sensitivity signals on the bilateral occipitoparietal lobes; (T2-6/7) high-sensitivity signals on the bilateral occipitoparietal lobes; (flair-1/2/3/4/5) high-sensitivity signals on the right parietal cortex and the bilateral subcortical white matter than T2.

Her doctors quickly consulted with ophthalmologists, neurologists, neurosurgeons, and physicians from the intensive care unit. After consultation, the chief doctor decided to perform brain computed tomography (CT) scan (Fig. 3) and magnetic resonance imaging (MRI) (Fig. 4). During the CT scanning and MRI, the patient slowly regained consciousness but was still unable to see. At 19:50, the skull CT scanning and MRI were completed. The neurosurgeon and neurologist discussed their diagnosis and suggestions for treatment according to their interpretation of the scans.

The CT shows high-density signals in the posterior fossa. The MRI–T2–flair shows high-sensitivity signals on multiple areas, mainly on bilateral occipitoparietal lobes, including the cortical and subcortical areas. Concurrently, diffusion-weighted imaging (DWI) showed low-sensitivity signals on the cortex of the bilateral occipitoparietal lobe. The T2 was same with the T2–flair, but was somewhat unclear.

### 1.4 Therapeutic interventions

Based on CT findings, subcortical hemorrhage could not be ruled out. The neurosurgeon suggested nimodipine to prevent cerebral vasospasm and mannitol and furosemide to relieve the cerebral edema; the ophthalmologist suggested methylprednisolone to prevent inflammation and edema; the neurologists suggested carbamazepine to prevent seizures; and the chief obstetric doctor suggested calcium chloride to correct hypocalcemia (1.99 mmol/L).

At 23:55 on January 12, the patient complained of headache, but no nausea, vomiting, or confusion developed, and nimodipine was discontinued. One and a half hours later (1:34, January 13), the patient developed a twitch in her limbs, experienced lockjaw, and then lapsed into unconsciousness. A total of 50 mg IV midazolam was transfused to control the epileptic seizures. The same situation occurred at 3:08 on January 13, and her doctor administered a venous injection of 50 mg midazolam. The third eclampsia then stopped.

At 7:00 on January 13, the patient was still dizzy. Visual acuity improved to light perception, and bilateral fundus did not show any abnormity.

On the fourth day of admission (January 15, 2017), her vision returned prepregnancy status.

**Figure 5:**
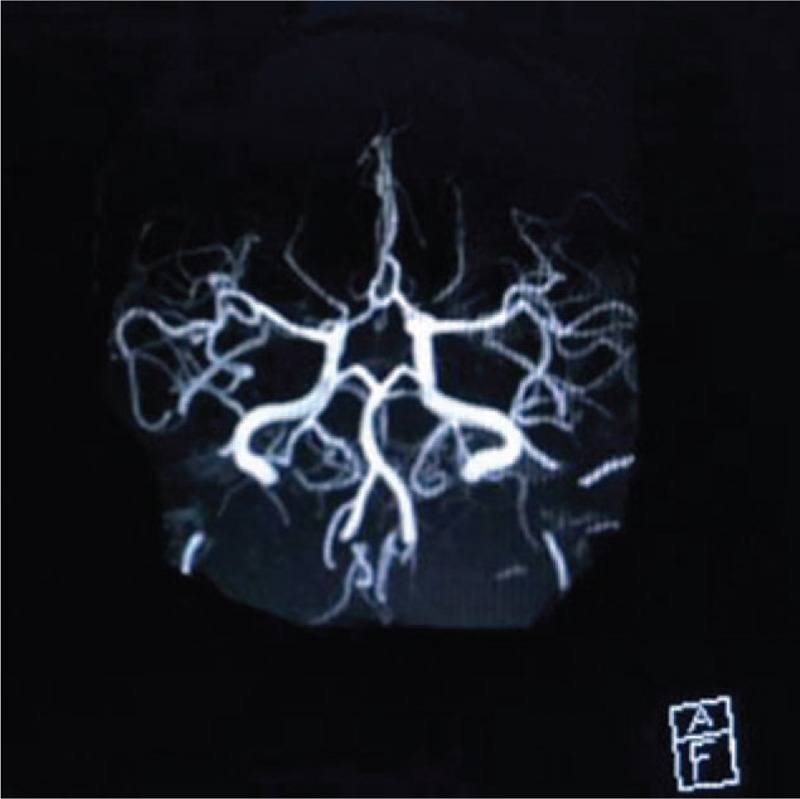
Normal cerebral blood vessels of the patient.

On the 11th day (January 22, 2017), she underwent head MRI, and the results were normal (Fig. 5).

Laboratory tests indicated her liver and renal function was normal. White blood cell count gradually increased and reached 20 × 10^9^/L. Urinal protein and type B natriuretic peptide were slightly higher than normal, whereas prothrombin time and activated partial thromboplastin time were normal.

The patient's vision normalized, and headache, nausea, and vomiting did not occur again. She was discharged after 13 days. However, her family did not understand her condition and filed for financial compensation. Thus, we did not follow the case owing to other accidental matters.

## 2 Discussion

### 2.1 Principal findings

After the first eclampsia, the CT scan showed high-density signals in the posterior fossa that looked like a subarachnoid hemorrhage (SAH) or cerebral venous sinus thrombosis (CVST). Because the main finding in the MRI–DWI and flair/T2 was bilateral occipitoparietal vasogenic edema, acute ischemia was ruled out.^[2]^ However, the high-density signals in the posterior fossa disappeared on the second CT scan on the next day. Thus, SAH or CVST was ruled out because the blood does not disappear within 24 hours, whereas contrast agents can be absorbed within 24 hours. Many reports showed that contrast staining was washed out within 24 to 48 hours, whereas hemorrhage persisted for days to weeks.^[15–16]^

The first and second eclampsia happened at 11.5 and 18.5 hours after admission, respectively. Blood pressure increased twice during the first 24 hours, and the timing of the 2 postpartum seizures coincided with the increase of the blood pressure. High blood pressure is associated with PRES.^[2]^ The eclampsia did not recur in the patient after the second day in the hospital. During that time, her blood pressure was gradually controlled to normality.

After timely and effectively supportive treatment, the patient finally recovered. The patient's vision normalized on the fourth day after admission to our hospital, and she no longer had epilepsy or complained of headache, nausea, or vomiting.

### 2.2 Why did she have PRES?

The patient's clinical and radiological features were consistent with PRES. PRES is characterized by a variable combination of headaches, seizures, altered mental status, visual impairment, focal neurological signs, and symmetric vasogenic edema in the bilateral posterior cerebral circulation region.^[2,17]^

However, the background of the patient is complex. In addition to the blood pressure fluctuation, she had postpartum hemorrhage, uterine artery angiography and embolization surgery, and postpartum eclampsia. Studies showed that PRES is associated with preeclampsia/eclampsia, hypertension, and sepsis.^[1,17–18]^ A recent case report showed that postpartum hemorrhage, without high blood pressure, can lead to PRES.^[14]^

Fisher and Schmutzhard^[17]^ reported that PRES may be related to blood–brain barrier (BBB) damage and vascular endothelial cell damage.^[17]^ Fugate and Rabinstein^[2]^ believed that although the pathophysiological changes underlying PRES are not fully understood, endothelial dysfunction is a key factor. Thus, we believe that blood pressure fluctuation, inflammation, and the use of contrast agents collectively caused BBB and endothelial cell damage, which eventually lead to PRES in this case.

Therefore, we concluded that that PRES was caused by multiple factors in this case. The risk factors for PRES include blood pressure fluctuation, inflammation, kidney dysfunction, cytotoxic drugs, and use of contrast agents, among others. Accordingly, patients with several of these conditions have higher risk for developing the disease.

### 2.3 PRES or CIE?

The background of the patient is complex. Aside from blood pressure fluctuation, she had postpartum hemorrhage and eclampsia and underwent uterine artery angiography and embolization surgery. Did the patient have PRES related to the use of iodine contrast agent or CIE?

Spina et al^[19]^ believed that the diagnosis of CIE require signs and symptoms of neurological dysfunction that are temporally attributable to cardiac catheterization, manifesting within minutes to hours of iodine-based contrast agent administration. These signs and symptoms completely disappear within 48 to 72 hours. Moreover, they are not attributable to other pathological processes such as stroke, seizure disorder, metabolic drugs, metabolic abnormalities, arterial dissection, and air embolization.^[19]^

CIE requires timely and effective treatment and prevention of thrombolysis. The prognosis of CIE is excellent with supportive management only.^[19]^ However, PRES might cause substantial morbidity and mortality that is often a result of acute hemorrhage or massive posterior fossa edema causing obstructive hydrocephalus or brainstem compression.^[2]^

Because CIE is still an exclusive diagnosis, we can only diagnose the patient as having PRES. However, the features of the 2 diseases are very similar, including clinical and radiological findings and treatment methods, except for prognosis.

### 2.4 Strengths and limitations

We explored the causes of PRES in this patient and found that CIE and PRES have many similarities. However, follow-up information was not obtained because of loss contact with the patient and her husband.
